# Advanced Sampling Methods for Multiscale Simulation of Disordered Proteins and Dynamic Interactions

**DOI:** 10.3390/biom11101416

**Published:** 2021-09-28

**Authors:** Xiping Gong, Yumeng Zhang, Jianhan Chen

**Affiliations:** 1 Department of Chemistry, University of Massachusetts Amherst, Amherst, MA 01003, USA; xipinggong@umass.edu (X.G.); yumzhang@umass.edu (Y.Z.); 2 Department of Biochemistry and Molecular Biology, University of Massachusetts Amherst, Amherst, MA 01003, USA

**Keywords:** conformational ensemble, enhanced sampling, generalized Born, Gō-model, implicit solvent, liquid-liquid phase transition, replica exchange, protein force fields

## Abstract

Intrinsically disordered proteins (IDPs) are highly prevalent and play important roles in biology and human diseases. It is now also recognized that many IDPs remain dynamic even in specific complexes and functional assemblies. Computer simulations are essential for deriving a molecular description of the disordered protein ensembles and dynamic interactions for a mechanistic understanding of IDPs in biology, diseases, and therapeutics. Here, we provide an in-depth review of recent advances in the multi-scale simulation of disordered protein states, with a particular emphasis on the development and application of advanced sampling techniques for studying IDPs. These techniques are critical for adequate sampling of the manifold functionally relevant conformational spaces of IDPs. Together with dramatically improved protein force fields, these advanced simulation approaches have achieved substantial success and demonstrated significant promise towards the quantitative and predictive modeling of IDPs and their dynamic interactions. We will also discuss important challenges remaining in the atomistic simulation of larger systems and how various coarse-grained approaches may help to bridge the remaining gaps in the accessible time- and length-scales of IDP simulations.

## 1. Introduction

Intrinsically disordered proteins (IDPs) or regions (IDRs), compared to well-structured proteins, do not have stable tertiary structures under physiological conditions. Nevertheless, IDPs or IDRs can be found in nearly a third of proteins encoded in the human proteome [1], and they play key roles in a variety of biological processes that underlie vital cellular functions ranging from signaling and regulation to transport [2,3]. The inherent thermodynamic instability of an IDP’s conformation allows it to respond sensitively to numerous stimuli, including binding, changes in cellular environments (e.g., pH), and post-translational modifications [4,5,6,7,8]. Such conformational plasticity arguably enables IDPs to interact with multiple signaling pathways and serve as scaffolds to form multi-protein complexes [9]. Importantly, IDPs and IDRs house around 25% of disease-associated missense mutations [10]. They have been considered promising therapeutic targets for treating various diseases (such as chronic diseases) [11,12,13]. While many IDPs have been shown to undergo binding-induced folding transitions upon specific binding [3], many examples are also emerging to demonstrate that IDPs can remain unstructured even in specific complexes and functional assemblies [14,15,16,17,18,19,20]. Such a dynamic mode of specific protein interactions seems much more prevalent than previously thought [21,22,23].

It is very challenging to provide reliable descriptions of the conformational ensembles of IDPs and IDRs. A disordered state does not lend itself to traditional structural determination methods that are geared toward describing a coherent set of similar structures. Biophysical techniques, such as NMR, SAXS, and FRET, can provide complementary information on various local and long-range structural organizations [7]. However, these ensemble-averaged measurements alone are not sufficient to unambiguously define the heterogeneous ensemble, due to the severely underdetermined nature of the structure calculation problem [8,24,25]. As a result, studies of IDPs have relied heavily in the traditional structure-function paradigm, by solving the folded structure of the bound state, analyzing coupled binding and folding mechanisms, or identifying putative pre-existing functional structures in the unbound state [3]. However, the disordered ensemble itself is arguably the central conduit of cellular signaling. The functional mechanism of an IDP is encoded in how the disordered ensemble as a whole responds to various stimuli, be it cooperative binding-induced folding or the redistribution of conformational sub-states in dynamic interactions. Multiple cellular signals can be naturally integrated through cooperative responses of the whole dynamic ensemble [26,27,28]. Therefore, there is a critical need for reliable characterization of disordered protein conformation ensembles, in both bound and unbound states, in order to establish the molecular basis of IDPs and IDRs in various physiological and pathophysiological processes.

**Figure 1:**
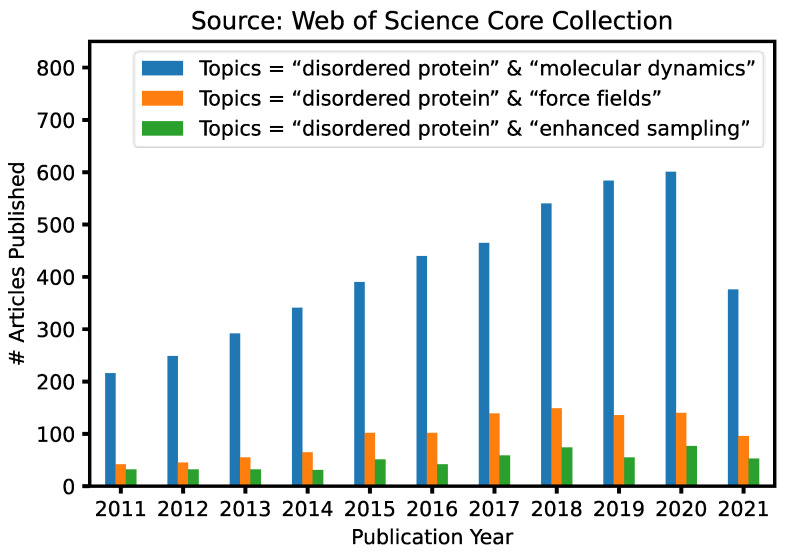
Number of articles identified with three different search keywords published from 2011 to 2021 based on a Web of Science core collection source (as of 15 August 2021).

Given the fundamental challenges of characterizing disordered protein states based on ensemble-averaged measurements alone, molecular modeling and simulations have a crucial and unique role to play in mechanistic studies of IDPs and IDRs [29,30,31,32,33]. This is reflected in continuously increasing numbers of research articles that contain keywords “intrinsically disordered” and “molecular dynamics” published in the last 10 years (Figure 1). A particularly attractive approach is to first generate the disordered ensemble using transferable, physics-based force fields without any experimental restraints and then use the later for independent validation [7]. Such *de novo* simulations of disordered protein ensembles require both high force field accuracy and adequate sampling of relevant conformational space, pushing the limit of these two central ingredients of molecular dynamics (MD) and Monte Carlo (MC) simulations. The challenges of simulating disordered proteins have driven significant interest in developing better protein force fields and advanced sampling methods (Figure 1). In particular, important advances have been made in the state-of-the-art atomistic force fields for describing the conformational equilibria of ordered and disordered proteins [13]. Enhanced sampling techniques have played crucial roles in both the development and application of atomistic force fields, by allowing one to cross energy barriers faster and accelerate the conformational sampling of IDPs [34,35,36,37,38,39,40,41]. Nonetheless, atomistic simulations still have limited capability in describing large systems such as biological condensates [42]. For this, multi-scale approaches are necessary to bridge the gaps in experimental and computational time- and length-scales, including implicit solvent models, which remove the solvent degrees of freedom [8], and various coarse-grained models, which significantly reduce both proteins and solvent degrees of freedom [43].

In this review, we will start by highlighting the challenges of sampling IDP conformational ensembles and providing a summary on the state-of-the-art force fields available to describe the IDP conformations. It is noted that several excellent review papers have been published recently that cover general theoretical and computational approaches for studying IDPs, in particular regarding protein–protein interactions and biological condensates [29,44,45,46]. This review will therefore focus on the recent development of advanced sampling methods for simulating disordered conformational ensembles and dynamic interactions of IDPs. We will also discuss some of the key advances in the multi-scale modeling of IDPs that greatly extend the accessible length- and time-scales of molecular simulations. Finally, we discuss future directions in developing a robust computational framework for simulating IDP conformational equilibria and interactions.

## 2. Challenges of Simulating IDP Conformational Equilibria

Compared to the globular proteins that have one or a few well-defined global energy minima, the energy landscape of an IDP is flatter and generally includes many local energy minima separated by modest energy barriers [47]. IDPs and IDRs typically have fewer hydrophobic residues, but a larger number of polar or charged as well as disorder-promoting residues (such as glycine and proline) [44]. These sequence features hamper the formation of hydrophobic cores that drive protein folding and thus prevent the formation of stable tertiary structures. Instead, IDPs and IDRs favor forming an ensemble of unfolded or partially folded states. This presents a major challenge for simulation and depends critically on the ability of the force fields to accurately describe the energetics of relevant conformational states, especially for capturing both folded and unfolded states of an IDP. For example, one recent study tested atomistic simulations of IDPs for eight force fields and found marked differences in the describing the conformational ensembles of IDPs, in particular the secondary structure content [48]. Similar observations have also been made in other benchmark studies, consistently showing that protein force fields previously optimized for folded proteins are not suitable for simulating disordered protein states, largely due to over-stabilization of protein-protein interactions [49]. These benchmark studies also suggested that the key towards better protein force field was to rebalance protein–protein, protein–water, and water–water interactions.

Besides accurate force fields, reliable simulation of IDPs also hinges on sufficient sampling of many relevant conformation states within a reasonable simulation time. Standard MD simulations are generally insufficient to generate representative conformational ensembles, even using the most accurate protein force fields coupled with advance of GPU computing or specialized hardware such as the ANTON supercomputer [50]. For example, a recent reanalysis of a 30-μs ANTON trajectory of a 40-residue Aβ40 peptide in explicit solvent revealed very limited convergence even at the secondary structure level [13]. This can be attributed to the diverse and large accessible conformational space of an IDP and the potentially high free energy barriers separating various sub-states that require exponentially longer time to cross. Note that typical simulation times on conventional hardware (such as GPUs) are at least one-order of magnitude shorter. There is thus great danger in relying on standard MD to calculate disordered protein conformational ensembles at the atomistic level. There is a critical need to develop and leverage so-called enhanced sampling techniques, which aim to generate statistically meaningful conformational ensembles with dramatically less computation. 

Computational studies of IDP interaction and assembly are even more demanding. The conformational equilibrium of an IDP can respond sensitively to specific and nonspecific binding, potentially shifting from a disordered to somewhat ordered state or fully folded state. In principle, simulations could provide the much-needed spatial and time resolutions to elucidate the kinetics and thermodynamics of coupled folding and binding processes and characterize the mechanistic features. However, the challenge is that this coupled process of folding and binding is a complex reaction involving the formation of many noncovalent interactions, which requires extremely long simulations generally beyond the current capabilities at the atomistic level. As such, coarse-grained models are generally required for computational studies of IDP interaction and assembly.

## 3. The State-of-the-Art Protein Force Fields for Describing IDP Conformations

Empirical protein force fields are potential energy functions that typically include physics-motivated bonded and non-bonded terms carefully parameterized based on a wide range of theoretical and experimental data [51]. These force fields can in principle be transferable between folded proteins and IDPs. To achieve this, it is also critical to develop suitable water models and better describe the water–protein interactions [52,53]. Two recent review articles have already provided comprehensive descriptions on the latest development of better protein force fields [51,54]. We therefore briefly summarize the state-of-the-art of nonpolarizable and polarizable force fields for IDP dynamics and interactions.

### 3.1. Nonpolarizable Protein Force Fields

Many previous nonpolarizable force fields have significant shortcomings for describing the unfolded or disordered proteins. For example, they typically provide a poor description of the secondary structure content for IDPs and have a preference to give too compact conformations with respect to the experimentally measured dimension of IDPs [48,55]. These problems were likely attributed to the unbalanced parameterization of dihedral torsion space and the description of protein–protein and protein–water interactions [56]. As a result, most of the improved force fields managed to give more accurate secondary structure propensities by adjusting dihedral parameters or adding grid-based energy correction map (CMAP) parameters [54]. The over-compactness of disordered proteins can be alleviated by modifying protein–water van der Waals interactions or combining with refined water models [52]. Representative state-of-the-art force fields includes the latest CHARMM36m/TIP3P* [57], ff19SB/OPC [58], and a99SB-disp/TIP4P-D [50]. Many benchmark studies have consistently demonstrated that these refined force fields do provide significant improvements in describing not only single folded and disordered proteins, but also the multiprotein systems that are either soluble or aggregate in the solution [55,59,60,61,62]. At the same time, these studies also identified significant remaining limitations in the description of the noncovalent interactions in the multiprotein systems [60]. Recognizing limitations in the ability of a99SB-disp/TIP4P-D force field to accurately describe the protein–protein interactions, a new force field, DES-Amber, was recently developed to provide more accurate simulations of protein–protein complexes while maintaining reliable descriptions of both ordered and disordered single-chain proteins [61]. However, DES-Amber is still limited in reproducing the experimental protein–protein association free energies of some protein complexes, in particular for the systems with highly polar interfaces [61]. In the latter case, it was found that the charged sidechains were buried at the protein–protein interface instead of being solvent-exposed. It was further suggested that nonpolarizable force fields were fundamentally limited in achieving a balanced description of charged groups that were solvent-exposed or buried at a protein–protein interface. 

### 3.2. Polarizable Protein Force Fields

Polarizable force fields explicitly consider the electronic polarization using various empirical models to provide better description of charged and polar protein motifs in heterogeneous biomolecular environments [63]. Exciting progress has been made in the last few years and several polarizable force fields are now available for the stable simulation of proteins in both aqueous and membrane environments [64,65]. Simulations using the latest polarizable force fields have also showed a high level of consistency with experimental observations, particularly the ion solvation and binding thermodynamics, permeation free energy of ions or small charged molecules into the cell membrane, and protein–ligand binding [63]. For example, the Drude-2013 polarizable force field, compared to CHARMM36 force field, is more accurate in describing the folding cooperativity of (AAQAA)_3_ peptide, which can be attributed to enhanced backbone dipole moments in the helix state [66]. Additional studies are still needed to show the necessity of considering polarizable force fields in IDP simulations, where the significantly higher computational cost adds to the challenge of generating converged ensembles [63]. Existing comparisons suggest that polarizable force fields, including AMOEBA and Drude models, still frequently have problems in reproducing the nature structures and folding of proteins [67,68,69]. For example, stronger protein–water interactions in polarizable force fields can destabilize the native protein structure, in opposition to the observations from nonpolarizable force fields where protein–water interactions have traditionally been underestimated [42]. Nonetheless, it can be anticipated that polarizable force fields will continue to be improved and become increasingly important for simulating IDP structure and interactions. 

## 4. Enhanced Sampling Methods for Sampling IDP Conformational Ensembles

**Figure 2:**
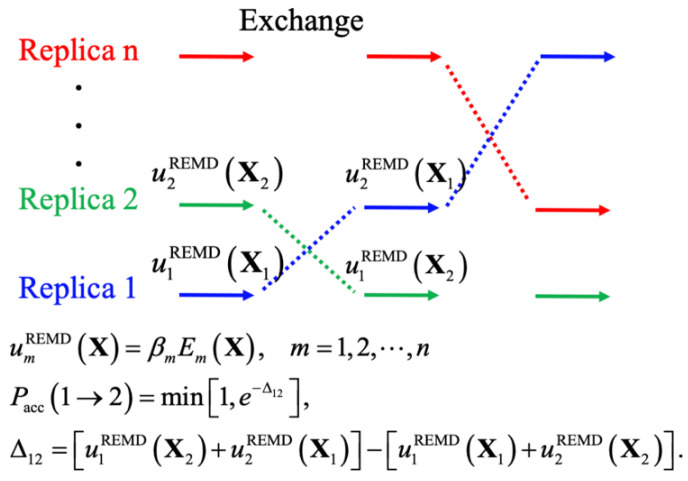
The generalized replica exchange molecular dynamics protocol based on unitless potentials, where the initial condition of each replica could have a varied temperature or scaled potential. *β_m_* is the inverse of temperature, *E_m_*(**X**) is the potential energy of m^th^ condition for given a configuration **X**.

Enhanced sampling techniques generally accelerate the crossing of energy barriers to achieve better sampling efficiency, such as by introducing bias potentials, modifying the potential energy itself, and changing the effective temperature. These techniques have proven essential in atomistic simulations of IDPs [70,71], yielding levels of convergence that could not be achieved even with drastically longer standard constant-temperature MD simulations [13]. The central idea of biased MD simulations is similar to importance sampling in MC simulations, where a biased potential is introduced to construct a flat free energy landscape along single or multiple collective variables of interest, such that many states can be readily sampled due to the removal of free energy barriers. The replica-exchange (REX) class of sampling methods, particularly replica exchange molecular dynamics (REMD), has been one of the most popular methods for simulating protein conformations. Figure 2 shows the general scheme of REMD simulations, where the key point is to first set up multiple replicas with different unitless unbiased or biased potentials, given as the energy over *k*_B_*T* (*T* is the temperature), and then use the Metropolis rule to allow MC to exchange the replicas and maintain the detailed balance. A key advantage of using multiple replicas and maintaining detailed balance is avoiding the reweighting problem generally required for biased simulations. Note that virtually all biased sampling strategies can be readily incorporated within the REX framework to benefit from both classes of enhanced sampling, including metadynamics (MTD) [72,73], accelerated MD (aMD) [74], umbrella sampling (US) [75,76], and integrated tempering sampling [77]. In practice, effective REMD protocols require a proper choice of (1) the optimal number of replicas and proper distributions of conditions, to ensure a uniform exchange acceptance rate and efficient random walk in the condition space, and (2) the choice of those unitless (biased) potentials for effective conformational diffusion at each condition [78]. Here, we divide various enhanced sampling strategies into two general groups depending on the need for collective variables and discuss their recent applications to IDP conformational sampling. These methods are summarized Table 1.

### 4.1. Collective Variables-Based Sampling Methods and Optimization

MTD and its variants have been considered one of the most important collective variables (CV)-based sampling methods for protein simulations [90]. MTD uses a history-dependent bias potential, which is generally a sum of Gaussians, to eventually construct a flat free energy landscape along the predetermined CV(s). A well-tempered MTD (WT-MTD) was later developed to increase the convergence, by gradually reducing the size of Gaussians based on the total accumulated bias potential [72,73]. Furthermore, the parallel tempering MTD (PT-MTD) and the combinations with other biased sampling methods have been also developed to increase the sampling efficiency and convergence of free energy calculations [91,92]. Representative examples include the PT-MTD that combines WT-MTD with PT or bias-exchange MTD that uses a different CV in each replica, rather than exchanging the temperatures. For example, the PT-WTD and bias-exchange MTD has been employed to obtain the conformational ensembles and coupled binding and folding of disordered pKID and KID proteins, using the α-score of helical structures as CVs [79]. It has also been shown that the REMD-based MTD, compared to conventional MTD or T-REMD, can enhance the conformational sampling of N-Glycans using dihedral angles as CVs to characterize the global motions [93]. The binding mechanism of two disordered peptides, NRF2 and PTMA, was simulated by the WT-MTD, and the results showed that the WT-MTD method could provide converged free energy profiles with 1.5 μs of sampling time [94]. Together, these applications have shown that MTD-class of sampling methods can be effectively applied to IDP simulations. Beside MTD, another important class of CV-based sampling strategy is the US method [76]. US is not strictly an enhanced sampling method like MTD. It typically uses multiple harmonic potentials to focus on sampling various states along the collective variables of interest. US is often combined with REMD in studies of IDPs, as illustrated in a recent 2D window-exchange US simulation of the coupled folding and binding mechanism of HdeA homodimer [80]. The simulation was able to capture rare unfolding transitions of the dimer at neutral pH and provided a detailed description of the transition pathways.

A central limitation of CV-based sampling methods is that the efficiency strongly depends on the quality of selected CV(s). For diffusion processes such as protein conformational fluctuation, it is often not clear which CVs can best capture large-scale transitions or even if these transitions could be effectively described using one or a few CVs [95,96,97]. Another practical limitation is that the computational cost of MTD and US grows exponentially as a function of the number of CVs, generally limiting the maximum to three. Parallel bias metadynamics (PBMetD) approaches have been proposed to overcome this limitation, by applying multiple low-dimension bias potentials in parallel [98,99]. Nonetheless, the efficacy of PBMetD for sampling complex (disordered) protein conformational space is yet to be demonstrated. Another recent work presented a temperature accelerated sliced sampling method to explore the high dimensional free energy landscape by combining Temperature-accelerated MD/driven-adiabatic free energy dynamics (TAMD/d-AFED), MTD and US methods to sample many CVs simultaneously [100]. However, the approach shares the limitation of PBMetD where the underlying bias potentials remain low dimensional in nature. To address the problem of determining the best CVs for a particular problem of interest, machine learning algorithms and deep learning network have been recently proposed to analyze information from many candidate CVs and construct the free energy landscape using low-dimensional representations [81,82]. On-the-fly discovery of optimal CV was also demonstrated using the artificial neural networks that has a strong capacity of learning and optimization for given linear or nonlinear CVs [101]. In another recent study, an eight-dimensional optimal biased potential was constructed and applied to the free energy calculations of polypeptides using two machine learning algorithms, namely the nearest neighbor density estimator and artificial neural network [102]. Similar deep neural networks have also shown to be capable of constructing nontrivial biased potentials, for deep enhanced sampling of protein conformational space and overcoming so-called hidden barriers [103,104]. These are exciting developments that may greatly expand the applicability of MTD, US, and other CV-based sampling techniques to problems of increasing complexity, including simulations of IDPs and their dynamic interactions, especially when combined with REX. 

### 4.2. Collective Variables-Free Sampling Methods and Optimization

CV-free sampling avoids the need to identify a set of optimal CVs and can be highly desirable for simulating high-dimensional conformational fluctuation of IDPs. Many CV-free sampling methods have also been developed, including the tempering-based and energy-scaled biased methods. Tempering-based sampling methods rely on increasing the effective simulation temperature (i.e., tempering) to accelerate barrier crossing. Examples include the temperature cool walking [105], annealed importance sampling [106], simulated tempering [83], and temperature-based REMD (T-REMD) [36]. T-REMD, in particular, has proven highly effective for protein folding and studies of IDP conformation ensembles, where multiple replicas are simulated at different temperatures in parallel to promote barrier crossing as the system undertakes a random walk in the temperature space (Figure 2). Nevertheless, one potential limitation is the number of replicas required for T-REMD scales, as the squared root of the number of degree of freedoms (DOFs) of the whole system, to maintain a reasonable exchange acceptance probability. This can dramatically increase the computational cost of the explicit solvent T-REMD simulations. Several methods have been proposed to avoid the demanding cost, such as adding energy-related terms (such as accelerated-MD or Gaussian accelerated MD, named GaMD) or scaling the potential energy function (including the scaled MD that scaled all energy terms and replica exchange solute tempering (REST) methods that scaled part of energy terms) [88,93,107,108].

aMD adds boost potentials to reduce the energy barriers and accelerate sampling [74]. However, it suffers from a serious energetic noise when reweighting [109]. The GaMD has been thus developed to reduce noise by introducing a new harmonic boost potential, to allow a new reweighting technique that could accurately recover the free energy landscape using a cumulant expansion to the second order [86]. GaMD has achieved some success in studying protein folding, protein–ligand binding, and protein–protein interactions [109]. In particular, specifically developed Ligand GaMD [110] and Peptide GaMD [111] can capture the binding and dissociation of molecular ligands and highly disordered peptides within microsecond simulations. Recently, this GaMD method has also been combined with the REMD protocol, which can avoid the energy reweighting problem [108]. A combination of replica-exchange umbrella sampling (REUS) and GaMD has also been designed for the conformational sampling and free energy calculations [88]. It is noted that the CVs-free enhanced sampling methods are more generally more suitable for simulating IDP conformations and dynamics, because of the difficulty of identifying appropriate CVs for IDP simulations as discussed above.

REST is a special variant of T-REMD designed specifically to reduce the number of DOFs that contribute to the Metropolis criteria of replica exchange, such that smaller number of replicas is needed [37,85]. The basic idea of REST is to separate the system into two ‘hot solute’ and ‘cold solvent’ regions. The ‘solvent’ could be actual water molecules but could also be any region of the system where no tempering is to be applied. This offers great flexibility in tailoring REST for a specific system of interest. Even more generally, the ‘solute’ region can be defined to include only a subset of interaction terms within the ‘solute’ region, such as dihedral-angle energy or Lennard–Jones energy term in the generalized REST (gREST) method [84]. Temperature-dependent factors are used to scale the ‘solute’–‘solute’ and ‘solute’–‘solvent’ interactions, while keeping the ‘solvent’–‘solvent’ interactions intact:

(1) $$\begin{array}{l} u_{m}^{\mathrm{REST}}\left(X\right)=\lambda_{m}^{\mathrm{pp}}E_{\mathrm{pp}}\left(X\right)+\lambda_{m}^{\mathrm{pw}}E_{\mathrm{pw}}\left(X\right)+\lambda_{m}^{\mathrm{ww}}E_{\mathrm{ww}}\left(X\right),\\ \begin{array}{cccc} \mathrm{REST}1: & \lambda_{m}^{\mathrm{pp}}=\beta_{m}, & \lambda_{m}^{\mathrm{pw}}=\frac{\beta_{0}+\beta_{m}}{2}, & \lambda_{m}^{\mathrm{ww}}=\beta_{0}, \end{array}\\ \begin{array}{cccc} \mathrm{REST}2: & \lambda_{m}^{\mathrm{pp}}=\beta_{m}, & \lambda_{m}^{\mathrm{pw}}=\sqrt{\beta_{0}\beta_{m}}, & \lambda_{m}^{\mathrm{ww}}=\beta_{0}, \end{array} \end{array}$$

where **X** is the conformational coordinates and *β*_m_ is the inverse of *k*_B_*T*_m_. The scaling of ‘solute’-‘solute’ interactions allows the ‘solute’ to be simulated with an effective temperature of *T*_m_ while maintaining the ‘solvent’ temperature at *T*_0_. As a result, the exchange acceptance probability will be independent of ‘solvent’–‘solvent’ interactions, which reduces the effective system size and requires fewer replicas to cover the same temperature range. A key open choice in REST is how the ‘solute’–‘solvent’ term is scaled (Equation (1)). Different solute–solute and solute–solvent scaling factors can strongly affect the ability of driving conformational transitions of the selected ‘solute’ region. A strong solute–solute interaction favors to compact the protein conformations, whereas a strong solute–solvent interaction prefers the disordered, solvent-exposed conformations. Different scaling schemes lead to very different characteristics of REST1 (original) and REST2 (revised) protocols (Equation (1)). High temperature conditions favor the unfolded conformations in REST1, while both folded and unfolded conformations were observed in REST2 model for the condition with the same effective ‘solute’ temperature. The reason for this is that REST2 was designed to have a weaker solute-solvent interactions to promote the sampling of folded conformations even at high temperatures [85]. While this could allow the sampling of reversible folding transitions at all temperatures in REST2, it could lead to conformational trapping, hampering the sampling of disordered conformations of IDPs. One important implication is that the performance of REST can be sensitive to the balance of protein–protein and protein–water interactions of a given protein force field. For example, Liu et al. showed that, while REST2 was highly effective in generating converged ensembles of 61-residue p53 N-terminal transactivation domain (TAD) using a99sb-disp, it completely failed to converge even with ~1 μs/replica in CHARMM36m and CHARMM36mw force fields [112]. Separate standard MD simulations reveal that p53-TAD can readily escape the apparent trapped conformations observed during REST2, suggesting that these traps arise due to the imbalance of scaled protein–protein, protein–water, and water–water interactions [112]. 

REST has proven to be one of the most reliable choices for enhanced sampling of protein folding and particularly disordered conformational ensembles [113,114]. Sugita and co-workers leveraged gREST to target the dihedral-angle energy term and successfully sampled folding transitions of beta-hairpins and Trp-cage in explicit water, using fewer replicas but covering wider conformational space compared to REST2 [84]. Walsh et al. applied REST to investigate n16N disordered peptide conformational ensembles [115]. The conformations obtained via REST methods showed a high consistency with NMR experimental data. Furthermore, REST are specifically appropriate in simulating IDRs as the disordered region can be targeted in REST without tempering the well-structured region (or water). Zhou and co-workers studied the disordered loop of Staphylococcus aureus sortase A (SrtA) to order transition upon binding to calcium [116]. Chen and Liu characterized Bcl-xL interfacial conformational dynamics in explicit solvent [117]. Both works directly showed that REST covered broader conformational spaces for intrinsically disordered regions and led to faster convergence compared to either standard MD or T-REMD simulations. REST simulations have also been successfully integrated with experiments to study how cancer-associated mutations and drug molecules may modulate the disordered ensembles of p53-TAD and Aβ peptides in recent years [118,119,120,121].

Despite the success of REST for CV-free enhanced sampling, it does not benefit from targeted acceleration along specific CVs that are known to be rate limiting. For this, REST (or REX in general) has been combined with CV-based enhanced sampling to maximize the efficiency of sampling the complex, high dimensional conformational space of proteins. Some of the examples are discussed in the sections above. Here, we note a couple additional recent examples. By integrating free energy perturbation (FEP) and REST methods, Abel et al. obtained more thorough samplings of different ligand conformations around the active site and realized relative binding affinity predictions [122]. Okamoto and co-workers have applied the REUS/REST two-dimensional replica-exchange method to predict two protein–ligand complex systems with the help of REST to weaken the solute–solvent interactions but improve the binding events and REUS to enhance the sampling along with the reaction coordinates [87]. 

Multiscale enhanced sampling (MSES) is yet another fascinating example of a CV-free enhanced sampling strategy. Protein folding and other cooperative transitions such as self-assembly are known to be dominated by entropy barriers, which renders tempering ineffective for driving faster transitions. Coupled with a lack of obvious CVs, sampling complex conformational transitions of IDPs and their interactions is challenging for both CV-based and REX-based CV-free methods. For this, an effective solution is to couple atomistic simulations with a coarse-grain (CG) model, such that one could benefit from both faster transitions of CG modeling and accuracy of atomistic force field [123]. A particularly attractive approach was first introduced by Kidera and coworkers, where restraint potentials were used to couple CG and atomistic conformational dynamics along “essential” DOFs shared by the two models [35]. The bias introduced by the coupling potential is removed using Hamiltonian REX (H-REX). Chen and coworkers further adapt the method to utilize topology-based CG models (see below), better coupling potential and advanced Hamiltonian/temperature REX (H/T REX) [34,124,125]. Coupling the CG and atomistic models using restraints is a key strength of these MSES protocols. It allows full control of the energetic impact of diverged structures at different resolutions, which improves exchange efficiency and provides superior scalability to large systems. MSES coupling also provides robust tolerance of CG defects by preventing the CG model from dictating the conformational dynamics. The efficacy of MSES has been illustrated using several systems. It was highly effective in simulate reversible transitions of small β-hairpins and helical IDPs [34,124,125] and proved instrumental in further refinement of a GBMV2 implicit solvent protein force field for both ordered and disordered peptides [126]. Very recently, MSES was also observed to be effective in sampling the cis–trans transitions of lutein by coupling the atomistic model with the Martini CG model [127]. Nonetheless, the application of MSES to larger and more complex proteins has proven more challenging than originally expected, apparently due to the difficulty in effective coupling of CG and atomistic conformational fluctuations of a larger protein.

Other tempering methods including integrated tempering and simulated tempering have also been combined with different biased potentials to enhance sampling [89,128]. For example, an integrated accelerated MD method has recently been used to sample the conformations of pepX peptides, and it was shown that this method can improve the sampling efficiency and provide a good strategy for simulating IDPs [69,89]. The combination with the metadynamics has also been presented to sample the conformational space of silica, and the acceleration was increased by over one order of magnitude [128]. One significant benefit is that only a single replica is required and could be suitable for Anton specialized hardware [50]. However, one drawback is that we have to estimate the relative free energies of all conditions (or equivalently the density of states), which requires recursive simulations and can be difficult to converge for complex systems, such as large IDPs and complexes.

### 4.3. Reweighting Techniques for Generating Unbiased Ensembles

When bias potentials are used to enhance sampling, reweighting is often required to obtain the unbiased samples and construct statistically optimal unbiased free energy surfaces. Two reweighting methods are widely used for this, including the weighted histogram analysis method (WHAM) for the biased simulations with specific CVs and a more general multistate Bennett acceptance ratio (MBAR) approach [129,130]. The stability of both WHAM and MBAR can be susceptible to large energetic fluctuations due to exponential dependence of weights on the value of the unitless potentials. Large energy fluctuations among sampled conformations can lead to large uncertainties during reweighting and thus final unbiased distributions. Another population based reweighting method has been used for unbiasing the scaled MD simulations by making a multidimensional histogram of all sampled configurations [131]. However, the dimensionality of configurational space is usually very huge, and thus can hardly be completely described by some dimensionality reduction techniques (such as principal component analysis). Recently, it was proposed that this energetic noise can be alleviated by truncating the cumulant expansion of the exponential average [86], which was originally used in the accelerated molecular dynamics. It has shown that it can accurately recover the free energy profiles within an acceptable error (~*k*_B_*T*), especially for the near-Gaussian biased unitless potentials [86]. This approximated reweighting methods have therefore been successfully used for reweighting several biased simulations [88]. It should be mentioned that those reweighting techniques can be used for reweighting any biased simulation, even for the REMD simulations. Nonetheless, all reweighting methods including MBAR relies on good overlap between the true conformational space and the region sampled by biased simulations. When the overlap is limited, the reweighted distributions will remain significantly different from the true result. The conformational space of even very short IDPs (e.g., ~10 residues or longer) can be complex enough to present formidable challenges for recovering the true disordered ensemble from a biased trajectory, generated either at high temperatures or with modified Hamiltonian. Instead of analyzing self-convergence (as a function of simulation time), a more rigorous test of convergence is to analyze results obtained from simulations initiated from distinct initial states (such as highly structured and fully disordered conformations [7]).

## 5. Multi-Scale Approaches for Overcoming Sampling Problems of Large Systems

As discussed above, dramatic improvement in atomistic protein force fields coupled with enhanced samplings and GPU computing have now enabled us to generate the disordered conformational ensembles of increasingly complex IDPs in both bound and unbound states. Many important phenomena related to IDPs remain largely out of the reach of physics-based atomistic simulations, such as aggregation [132,133,134] and biological condensates [135,136,137,138]. Here, we review two of the key multi-scale approaches that allow one to simulate longer time-scale bioprocesses and more complex systems within the capacity of current computational capability, namely implicit solvent and coarse-grained (CG) models. Both approaches have been extensively studied and applied to globular proteins as well as IDPs. 

### 5.1. Implicit Solvent Models for Removing Solvent DOFs

Implicit treatment of solvent is an effective approach to reduce the computational cost of atomistic IDP simulations. The basic idea is to directly estimate the solvation free energy to capture the mean effect of solvent on the thermodynamic properties of the solute [139]. Implicit solvent is essentially a multi-scale model, where the solvent is represented using certain physical model while keeping atomistic details of the solute. These models have emerged as attractive alternatives for simulations of IDPs and their interactions compared to explicit solvent. In particular, many generalized Born (GB) based implicit solvent models have been developed, including the fast analytical continuum treatment of solvation (FACTS) [140], Amber GB models (such as GB-HCT [141], GB-OBC [142], and GB-Neck [143,144]), analytical generalized Born plus nonpolar (AGBNP) [145,146], and GB models implemented in CHARMM program (such as GBSW [147] and GBMV [148,149]). Several of these GB models can be optimized to provide a balance between computational efficiency and accuracy desired for IDP simulations [126,150,151], by systematic optimization of key physical parameters such as atomic radii to balance solvation and intramolecular interactions. Applied to various model IDPs with extensive experimental data, implicit solvent simulations have provided important insights on detailed conformational properties of the unbound state and how these properties may support function [32,33,152,153,154]. 

Despite many successes, implicit solvent models have not widely been tested and applied to the studies of larger IDPs. Several factors likely contribute to this. Most implicit solvent models are built upon existing protein force fields, which until recent years have had significant limitations in describing disordered protein conformations. Implicit treatment of solvent also relies on various approximations for computational efficiency, such as treating water as a continuous dielectric medium in GB models, limiting the ability of implicit solvent to accurately capture the conformational dependence of solvation free energy. A particular limitation is the common use of a surface area (SA)-based model to describe nonpolar solvation energy, which has known limitations in describing the length-scale dependence as well as solvent screening of dispersion interactions [151]. These limitations can result in a systematic bias towards an overly compact conformational ensemble, which is more pronounced for larger IDPs.

Several recent efforts have been made to further improve implicit solvent models for IDP simulations. The GB-Neck2 model has been optimized to reproduce solvation energies for a variety of protein systems [144]. Recent benchmark studies have shown that the GB-Neck2 model can reasonably discriminate folded and disordered peptides and could be used for quantitative protein folding simulations up to millisecond time scales [155,156,157]. Recently, the GBMV2 model, which includes an analytical approximation of molecular volume and is arguably one of the best GB models, has been implemented on the CUDA platform using the CHARMM/OpenMM interface [158]. The ~2 order of magnitude GPU acceleration greatly enables GBMV2 to simulate the conformation and interaction of larger IDPs. The ABSINTH implicit solvent model focuses on recapitulating the polymer properties of peptides and has been successfully used for a variety of IDP simulations, including Aβ peptides and the aggregation of phenylalanine [159,160] and sequence-conformation relationship of IDPs in general [8,161]. Recently, an ABSINTH-C model was developed to address the problem of overly shallow Ramachandran distributions of ABSINTH, by adding residue-specific correction terms [162]. The new model not only has a capacity to maintain stable native structures of α-/β-folded proteins, but also to increase the reversible folding of β-hairpin peptides.

### 5.2. Coarse-Grain Models for Reducing the DOFs of Proteins

**Figure 3:**
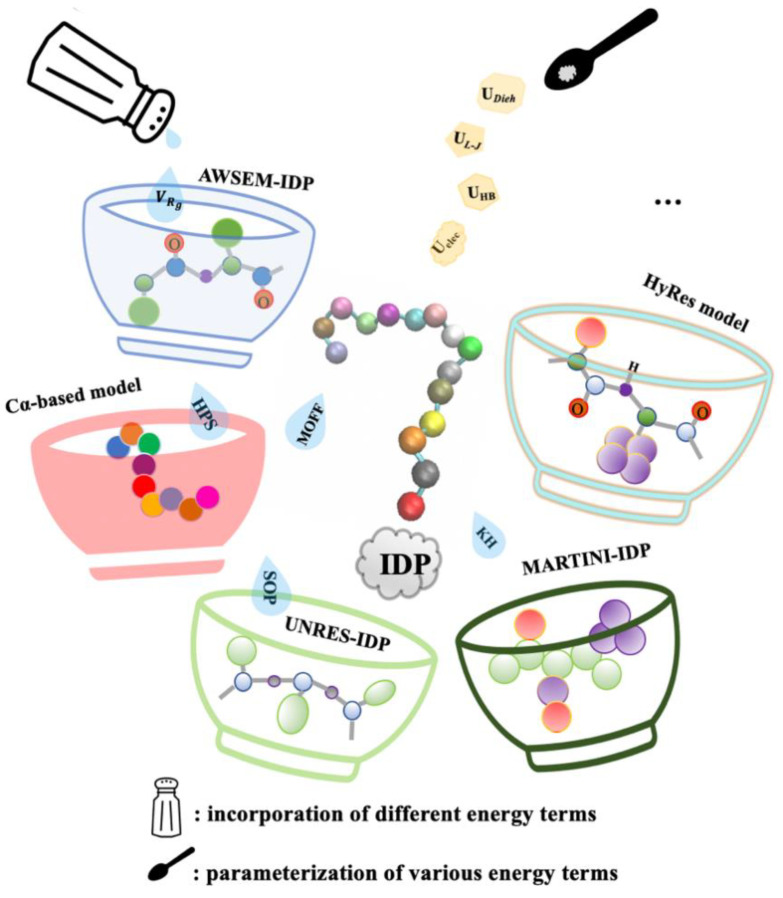
Coarse-grain modeling for addressing various IDPs-related challenges. These models can have a range of spatial resolutions and may be refined by introducing various effective potentials and/or re-calibrating the parameters of these energy terms.

Notwithstanding the ever-improving atomistic modeling, coarse-graining has remained an attractive and often effective strategy for extending the accessible time and length-scales of MD simulations. By grouping multiple (protein) atoms into CG beads and using simplified potential energy functions, CG modeling does not only reduce the system size, often by ~10-fold, but also allows much larger MD integration time steps up to 10 s of fs. Together, many CG models can be several orders of magnitude more efficient than atomistic ones. Numerous CG models have achieved varying levels of success in studies of protein folding, binding, and assembly [43,163]. Nonetheless, there are important distinctions between the conformational properties between globular proteins and IDPs, as well as the relative importance of electrostatic, hydrophobic, and hydrogen-bonding interactions in governing their conformational equilibria. Therefore, CG models optimized for the folded proteins are generally not suitable for the IDP simulations. It is often necessary to readjust the parameters of protein–protein and protein–solvent interactions or add new terms for more accurate description of IDP conformations (Figure 3). Here, we summarize several of these refined CG models for more efficient sampling of IDP conformation and interactions as well as their successes and limitations.

Gō/Gō-like models, also known as topology-based models, are based on the funneled energy landscape theory [164] and have been highly successful in describing the folding mechanism and pathway of structured proteins [165]. Somewhat surprisingly, Gō-like models have also proven effective for determining the mechanism and kinetics of IDP interactions, particularly the coupled binding and folding process [166,167,168,169,170,171]. The implication is that the binding and folding are governed by similar principles that require minimal frustration for efficiency. Note that Gō-like models generally require additional calibrations to provide a more quantitative description of the balance between intermolecular interactions and intrinsic conformational propensities [172]. A key limitation of the topology-based modeling of IDPs is lack of the ability to capture the impacts of non-“native” structural features and nonspecific interactions, which could play important roles in IDP structure and function. This may be partially overcome by including new energy terms (Figure 3), such as explicit charge-charge interactions, inert crowder molecules, and confinement potentials. A particularly interesting discovery from such extended topology-based modeling of IDPs is the role of long-range electrostatic interactions in promoting efficient coupled binding and folding, allowing IDPs to fold at timescales beyond the μs “folding speed limit” to avoid a potential kinetic bottleneck in specific recognition [167,169,173]. IDP-binding proteins have evolved to contain charges near the binding interface to complement those highly conserved charges on IDPs. Long-range electrostatic interactions between these charges do not only accelerate the encountering of IDPs, but also promote the efficiency of IDP folding upon nonspecific encounters.

Several higher resolution coarse-grained models have been also developed specifically for modeling IDPs. Thirumalai and co-workers reparametrize the two-bead self-organized polymer coarse-grained model (SOP-CG) to reproduce Rg values of a set of diverse IDPs with 20 to 441 residues [174]. The resulting SOP-IDP also accurately reproduce the small-angle X-ray scattering profiles for these IDPs. Nonetheless, SOP-IDP is designed for IDPs solely and lacks transferability and compatibility in describing even small globular proteins under physiological conditions. Recognizing the limitation of C⍺-only backbone representation in capturing the intrinsic conformational propensities of IDPs, Chen and Liu developed a hybrid resolution (HyRes) model that contains an atomistic description of the backbone, to provide a semi-qualitative description of the secondary structure propensities, and intermediate resolution side chains, to allow qualitative description of the overall peptide chain dimension and transient long-range interactions [175]. While HyRes was originally designed for driving faster atomistic sampling for MSES simulations, applications to a set of small and large IDPs including p53-TAD suggest that HyRes may be appropriate for simulating IDP structure and interactions by itself [175]. Papoian and co-workers have developed the AWSEM-IDP model that can be used to efficiently sample the large conformational space of IDPs and at the same time can distinguish the levels of peptide chain expansion of globular proteins and IDPs [176]. AWSEM-IDP includes only C_⍺_, C_β_, and O atoms, and has been reparametrized for IDPs by adjusting the secondary structure-related potential energy terms as well as introducing a new parameter, *V*_Rg_ term, for controlling the collapse and size fluctuation of the protein. 

An important application for CG models is to study liquid-liquid phase transitions (LLPS) that are frequently mediated by IDPs [29,44,45,46]. Dignon et al. proposed a residue-based C_⍺_-only CG model to represent the disordered low complexity domain of the RNA-binding protein FUS-LCD and the DEAD-box helicase protein LAF-1 in the formation of LLPS [177]. The model uses the Debye–Hückel approximation for long-range electrostatic interactions and the hydrophobicity scale model [177] or the Kim–Hummer model [178] for short-range residue–residue interactions. The results indicated that both two approaches could reproduce the experimentally observed phase behaviors and changes in phase diagrams caused by mutation. Although they mentioned that the temperature-dependent phase behaviors were not compatible with the experimental absolute temperature and the ionic strength dependence was not fully tested due to the breakdown of the Debye-Hückel electrostatic energy potentials. The model could be further refined. For example, more residue-type parameters were considered to account for phosphorylation and acetylation effects [179], which allows in-depth investigation of how post-translational modifications may control LLPS behaviors. Recently, Latham and Zhang re-tuned the model of Dignon et al. to better reproduce the Rg distributions of a set of folded and disordered proteins [180]. The resulting maximum entropy optimized force field (MOFF) includes a new residue–residue interaction matrix and is more transferable for modeling both globular proteins and IDPs. Hummer and co-workers modified the MARTINI model via re-scaling the solute–solute non-bonded Lennard–Jones potentials to reproduce the experimental transfer free energies of phase separation among dilute and dense liquid phases and proposed a more general approach in tuning CG models with MD for LLPS related studies by optimizing and balancing the solute–solute and solute–solvent interactions, then matching the CG data to the atomistic simulation or experimental results [177]. The resulting MARTINI-IDP model was shown to successfully simulate the droplet formation and capture reversible phase transformations. Such exciting progress highlights the strong potential for simple C_⍺_-only CG models in molecular simulations of LLPS involving IDPs. Nonetheless, difficulty in describing local structure propensities (such as transient helices) with the C_⍺_-only representation may be an important limitation for studying certain specific effects of IDPs in LLPS. 

## 6. Concluding Remarks

Effective and reliable molecular simulations are crucial for characterizing the details of disordered conformational ensembles of IDPs in isolation, dynamic complexes, or biological condensates. Such computational capability, integrated with experimental studies, makes it possible to determine how the dynamic protein states may respond various cellular stimuli in signaling and regulation and more rigorously establish the (dynamic) structure-function relationship of IDPs and IDRs. In this review, we highlight recent advances in meeting two central requirements for reliable IDP simulations, namely accurate force fields, for describing the energetics of protein conformations, and efficient MD simulation methods, for the adequate sampling of relevant conformational space. The need to simulate disordered protein ensembles has played a key role in driving significant improvements in empirical protein force fields in recent years. Many of these force fields are now well balanced for both folded and disordered proteins. The force field development itself has directly benefited from many advanced sampling methods that allow for accurate calculation of the conformational equilibria of model peptides and proteins during force field recalibration. These enhanced sampling techniques rely on carefully designed biasing potentials, modification to the original Hamiltonian, and/or tempering to accelerate barrier crossing and generate statistically meaningful ensembles with far less computation. Many of the enhanced sampling strategies are complementary and can be readily integrated together to further improve the efficiency. Together, the improved protein force fields and powerful sampling techniques now allow realistic simulations of the conformation and interaction of at least modest-sized IDPs at the atomistic level. 

Nonetheless, the high dimensionality and complex nature of disordered protein conformation continues to push the limits of the force field and sampling capability. In particular, none of these methods alone appears to be generally applicable to simulate IDPs that are large (e.g., more than a few dozens of residues) and/or contain nontrivial residual structural features. There remains an urgent need and exciting opportunities in developing much more effective methods for sampling IDP conformations and dynamic interactions, such as through the careful integration of various existing CV-dependent and CV-free strategies. A particular promising direction is to leverage machine learning to design superior adaptive sampling strategies that can generate optimal bias potentials on the fly to maximally drive the exploration of the free energy landscape. 

Many proteins models with various levels of resolution are also being developed and fine-tuned for IDP simulations, particularly for studying biological condensates. These models range from C_⍺_-only single-bead protein models to implicit solvent ones with atomistic proteins. Many of the current models are geared towards modeling systems with minimal residual structures. A key challenge in the multi-scale modeling and simulation of IDPs is finding the optimal compromise between resolution, accuracy, and efficiency for the particular problem of interest. Nonetheless, it can be expected that multi-scale simulations will continue to play a central role in studying IDPs and dynamic interactions. 

## Figures and Tables

**Table 1:** Summary of enhanced sampling methods for IDP simulations.

Types	Sampling Methods	Key Features	References
CV-based	WT-MTD	History-based adaptive bias potentials	[72,73]
	Bias-exchange MTD	Multiple replicas with bias on different CVs	[79]
	Umbrella sampling	Pre-determined bias potentials	[80]
	Machine learning	On-the-fly discover optimal CVs	[81,82]
Tempering-based	Simulated tempering	Random walk in the temperature space	[83]
	Parallel tempering	Multiple replicas to avoid the need for estimating the density of states	[36]
	Integrated tempering	Integral of Boltzmann distributions over a range of temperatures as the bias	[77]
	Solute tempering	Scaling the energies of only selected atoms or terms to achieve effective tempering	[37,84,85]
Accelerated MD	GaMD	Boost potentials to accelerate barrier crossing	[86]
Combinations	MSES	Temperature/Hamiltonian replica exchange simulation by coupling CG and atomistic models	[34]
	REUS/REST	Combined REUS and REST	[87]
	REUS/GaMD	Combined REUS and GaMD	[88]
	Integrated aMD	Integrated aMD and integrated tempering	[69,89]
	PT-MTD	Combined the WT-MTD with PT	[79]

## Data Availability

Not applicable.
